# Prediction-based approaches to characterize bidirectional promoters in the mammalian genome

**DOI:** 10.1186/1471-2164-9-S1-S2

**Published:** 2008-03-20

**Authors:** Mary Qu Yang, Laura L Elnitski

**Affiliations:** 1 National Human Genome Research Institute, National Institutes of Health, US Department of Health and Human Services, Bethesda, MD 20892, USA

## Abstract

**Background:**

Machine learning approaches are emerging as a way to discriminate various classes of functional elements. Previous attempts to create Regulatory Potential (RP) scores to discriminate functional DNA from nonfunctional DNA included using Markov models trained to identify sequences from promoters and enhancers from ancestral repeats. We proposed that knowledge gleaned from those methods could be further refined using a multiple class predictor to separate classes of promoter elements from enhancers or nonfunctional DNA.

**Results:**

We extended our previous work, which identified over 5,000 candidate bidirectional promoters in the human genome, to map the orthologous promoter regions in the mouse genome. Our algorithm measured the robustness of evidence provided by the spliced EST annotations and incorporated evidence from annotations of UCSC Known Genes and GenBank mRNA. In preparation for de novo prediction of this promoter type, we examined characteristic features of the dataset as a whole. For instance, bidirectional promoters score very highly among all functional elements for Regulatory Potential Scores. This result was unexpected due to the limited sequence conservation found in these noncoding regions. We demonstrate that bidirectional promoters can be classified apart from other genomic features including non-bidirectional promoters, i.e. those promoters having no nearby upstream genes. Furthermore bidirectional promoters consistently score at the level of very highly conserved functional elements in the genome- developmental enhancers. The high scores are due to sequence-based characteristics within the promoters, not the surrounding exons. These results indicate that high-scoring RP regions can be deconvoluted into various functional classes of genomic elements. Using a multiple class predictor we are able to discriminate bidirectional promoters from enhancers, non-bidirectional promoters, and non-promoter regions on the basis of RP scores and CpG islands.

**Conclusions:**

We examine orthology at bidirectional promoters, use discriminatory machine learning approaches to differentiate multiple types of promoters from other functional and nonfunctional features in the genome and begin the process of deconvoluting classes of functional regions that score well with RP scores. These types of approaches precede supervised learning techniques to discover unannotated promoter regions.

## Background

The intricate details of regulated gene expression are not well-characterized in the human genome. Currently our understanding relies greatly on our ability to experimentally identify prospective regulatory regions and to computationally evaluate features of those experimental datasets. We have found that searching for genes arranged in a ‘head-to-head’ configuration can precisely identify a set of candidate regulatory regions, without the intermediate step of experimental identification. The designation of the 5′ and 3′ ends of a gene (i.e. from start-to-stop or head-to-tail) indicates that a head-to-head arrangement places the transcription start sites (TSSs) of two genes in close proximity. The directionality of transcription (from 5′ to 3′) by RNA polymerase allows these adjacent genes to produce products without interfering with each other. Two genes in a head-to-head configuration that have their 5′ ends located fairly close together, within 1000 base pairs, are assumed to have a shared promoter region located between the two 5′ ends. This promoter is defined as a bidirectional promoter, because it influences expression of the two genes simultaneously. This influence can be concordant or discordant.

Bidirectional promoters occur frequently in the human genome [1-3]. Despite their prevalence, their full biological significance is not yet known. Nevertheless, evidence of significant biological implications is emerging [4]. Further elucidation may come from studies in other species' genomes. The process of mapping bidirectional promoters in other species is fairly simple once the algorithms are developed. More importantly, a comprehensive set of these regulators in multiple species allows comparative analyses across species. Predictions made within a single species can be validated by their appearance in another. Bidirectional promoters represent a special class of promoter sequences, specifically those having an exon on either side of the promoter region (i.e. the first exon of each gene regulated by the promoter). Thus, the promoter region is ‘bounded’ by sequences with described functions on both sides, and thereby limited to the intervening portion. This arrangement solves the problem of defining the upstream boundary of the promoter, which is a troublesome reality of studying promoters with no discernible upstream endpoints. If fundamental differences are present in the sequences underlying functional elements, machine-learning approaches may be able to identify them. The key to success lies in a precise description of each of the functional categories. For instance, sequences characterizing bidirectional promoters can be compared to non-promoter regions found between the ‘tails’ of adjacent genes arranged in a tail-to-tail configuration. Additionally, further characterization may be possible by discriminating bidirectional promoter sequences from enhancer regions, which are often highly conserved and can act at extreme distances from a responsive gene. The most challenging regions to distinguish from bidirectional promoters are other promoter regions, including unidirectional promoters that have a neighboring gene (head-to-tail arrangement) and unbounded promoters, which have no upstream neighboring gene.

Progress in discerning classes of functional elements from each other, without the aid of experimental data, represents a significant goal in our ability to decode the human genome. In this manuscript, we present a detailed mapping of bidirectional promoters in the mouse genome, analogous to our work in the human genome [3]. Furthermore, we compare data from human and mouse as a means to validate our predictions, and to further characterize features within bidirectional promoters. Using bidirectional promoters as a model dataset, we describe results of machine learning approaches to score functional elements in genomic sequences. We conclude with a multiple class predictor that aims to accurately discriminate classes of promoters from one another, from enhancers, and from nonfunctional regions.

## Results and Discussion

### Mapping bidirectional promoters in the mouse genome

In an analogous approach to our studies in the human genome, we systematically mapped bidirectional promoters in the mouse genome. These promoters were defined by their position between two oppositely-oriented transcription units, whose transcription start sites (TSSs) were no more than 1000 bp apart. All transcripts used in the analysis originated at one of three repositories :

• The UCSC List of Known Genes [5].

• GenBank mRNA data [6].

• Spliced EST data from the GenBank dbEST database [6].

As discussed in [3] the procedure for mapping bidirectional promoters from the Known Gene annotations is quite straightforward due to the quality of these gene descriptions. Initially, all genes are represented as clusters containing overlapping transcripts. Each cluster extends from the farthest 5′ to the farthest 3′ coordinate of any included transcript. Neighboring clusters are then examined with respect to the distance and orientation of their 5′ ends. If the 5′ ends of two genes are no more than 1000 bp apart and the genes are transcribed in opposite directions, the region between them is considered to be a bidirectional promoter. Identifying bidirectional promoters from other annotation sources in the mouse genome can be more complex due to the diversity and fragmented nature of the current transcripts. For instance, both the spliced ESTs and the GenBank mRNA transcripts contain multiple overlapping segments of transcribed regions, which are frequently updated as new information becomes available. To handle the complexity of the data in the spliced ESTs, we applied an algorithm to extract the bidirectional promoters that passed a variety of conditional tests. These included conformity to the rules of distance and orientation.

Furthermore, transcripts were classified as intergenic or intragenic by comparison with the Known Genes as a reference track. Additional criteria requiring majority agreement with the orientation of co-localized ESTs and with the orientation of Known Genes are described in Yang and Elnitski (2007) [3].

**Figure 1:**
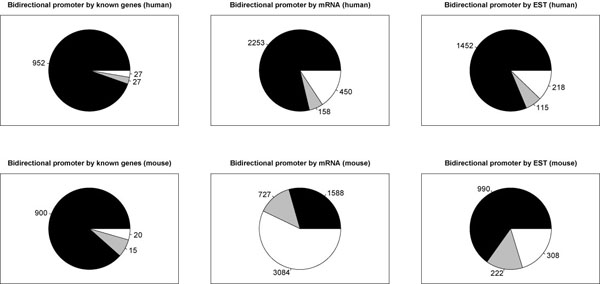
Validation of bidirectional promoters using the RIKEN CAGE dataset. Pie charts depict the number of bidirectional promoters with CAGE transcripts that correspond to detectable transcripts on both sides (black), only one side (gray), or no evidence (white). Note that these do not have to be transcribed in the same tissues to be included in our study. The upper panel is based on human transcripts from the human sequence assembly, hg17, while the lower panel uses CAGE data and transcripts from the mouse sequence assembly, mm5. Bidirectional promoters were mapped in Known Genes (left column), GenBank mRNA (middle column), and spliced ESTs (right column).

The mapping algorithm identified 5,647 candidate bidirectional promoter regions in the mouse genome. This number is similar to the number of candidate bidirectional promoters identified in the human genome using a similar strategy [3]. In both genomes, the number of bidirectional promoters was larger than previously reported [1,2], as a result of updated gene annotations and the use of spliced EST data. The validity of these candidate regions was assessed by comparison to the RIKEN CAGE dataset [7]. The CAGE technique captures the true 5′ ends of transcripts, allowing a direct comparison to our bidirectional promoters by their coordinates in the mouse genome. Figure 1 shows bidirectional promoters that are fully validated when a CAGE transcript flanks both sides of the promoter region. In the human genome, bidirectional promoters from the Known Gene, mRNA, and EST data are validated at 96%, 78%, and 81%, respectively (Figure 1, upper panel), while in the mouse genome, bidirectional promoters from the Known Gene, mRNA, and EST data are validated at 95%, 40%, and 65%, respectively (Figure 1, lower panel). The low validation score for mouse mRNA appears to reflect an incomplete description of the mouse genes in the mouse genome assembly mm5 (May 2004).

### Comparison of human and mouse bidirectional promoter sets

**Figure 2:**
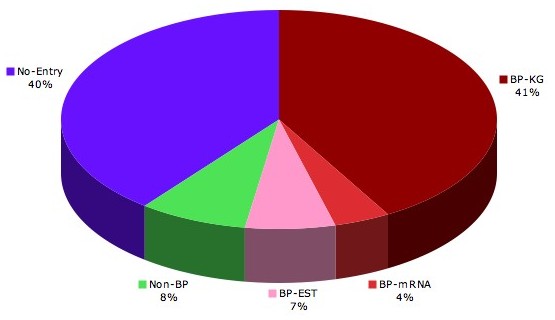
Orthologous mapping of human bidirectional promoters to mouse. Promoter orthology was de-termined by identifying ortholgous genes in mouse and checking for evidence of bidirectional promoters. Genes that had a 5′ neighbor transcribed in the opposite direction are shown for promoters of Known Genes(maroon), Genbank mRNA (pink), and ESTs (red). Genes with no neighbor in mouse lack evidence for bidirectional promoters (green). Genes that could not be mapped to mouse are shown in blue.

Bidirectional promoters are ancient features, exhibiting orthology from human to *Fugu rubripes*[8]. To compare the co-occurrence of bidirectional promoters in the human and mouse genomes, we mapped human genes regulated by bidirectional promoters to the mouse genome and assessed whether the corresponding mouse gene also formed a bidirectional promoter with its 5′ neighbor. Of 1637 Known Genes, as shown in Figure 2, 41% were associated with bidirectional promoters in the mouse genome by the same gene name. An additional 4% were added from Genbank mRNA and 7% from the spliced ESTs. Roughly 7% of the set had a gene in the mouse genome but shows no evidence of a bidirectional promoter. The remaining 40% could not be mapped to the mouse using this method. Table 1 shows the orthologous pairs of mouse genes corresponding to ten human genes involved in cancer that have bidirectional promoters. From this data we predict that 4 mouse genes will be positioned closer together than they currently appear. BRCA2, ERBB2, FANCA and FANCF are much farther apart in mouse than in human. Table 2 shows the GO terms for genes that are regulated by bidirectional promoters in human, but not in mouse, implying that regulatory changes could change the expression of these genes between species. It should be noted that strategies such as ours to map orthologs by gene name provide high confidence assignments, but underestimate the number of orthologous bidirectional promoters in the human and mouse genomes. We have further proven this point by mapping orthologous gene pairs regulated by bidirectional promoters in twelve species using rigorous genomic alignment information [9].

**Table 1:** Tumor suppressor genes in human and mouse

BOC gene	Bidirectional partner	Annotation of partner	Distance between TSSs	CpG island at TSSs
BARD1 (Human)	DA865307	mRNA, EST	518	Across/First exon of both
BARD1 (Mouse)	AK007117	mRNA	-425	Across/First exon of both
BRCA1 (Human)	NBR2	KG, mRNA, EST	81	Inside NBR2
BRCA1 (Mouse)	NBR1	KG, mRNA, EST	259	No CpG
BRCA2 (Human)	DR731263	EST	955	Overlaps First Exon of BRCA2
BRCA2 (Mouse)	CO801197	EST	2505	Overlaps First Exon of BRCA2
CHK2 (Human)	HSC20	KG, EST	32	Overlaps First Exon of both
CHK2 (Mouse)	AW049829	KG, mRNA, EST	276	Across/First exon of both
ERBB2 (Human)	Perld1	KG, mRNA, EST	60	Overlaps First Exon of both
ERBB2 (Mouse)	Perld1	KG, mRNA, EST	11,994	CpG at first exon of both
P53 (Human)	AK001247	KG, mRNA, EST	491	Overlaps First Exon of P53 Partner
P53 (Mouse)	WDR79	KG, mRNA, EST	657	Across/First exon of both
FANCA (Human)	Spisre2	mRNA, EST	1,533	Overlaps First Exon of both
FANCA (Mouse)	Spisre2	KG, mRNA, EST	14,137	CpG at first exon of both
FANCB (Human)	MOSPD2	KG, mRNA, EST	372	Across/First exon of both
FANCB (Mouse)	AK035985	KG, mRNA, EST	257	No CpG
FANCD2 (Human)	BC043599	KG, mRNA, EST	64	Across/First exon of both
FANCD2 (Mouse)	Tmem111	KG, mRNA, EST	47	Across/First exon of both
FANCF (Human)	GAS2	mRNA, EST	-199	Across/First exon of both
FANCF (Mouse)	AK014509	mRNA	1,966	Overlaps First Exon of FANCF partner

**Table 2:** Molecular function (*P* < 0.05) of human genes having a unique bidirectional promoter not detected in mouse

Go ID	Molecular Function
GO:0004004	ATP-dependent RNA helicase activity
GO:0008186	RNA-dependent adenosinetriphosphatase
GO:0047804	ATP-dependent RNA helicase activity
GO:0004042	N-acetylglutamate synthase activity
GO:0019145	aminobutyraldehyde dehydrogenase activity
GO:0000250	oxidosqualene-lanosterol cyclase activity
GO:0008321	Ral guanyl-nucleotide exchange factor activity
GO:0031559	oxidosqualene cyclase activity
GO:0047316	glutamine-phenylpyruvate aminotransferase activity
GO:0008176	tRNA (guanine-N7-)-methyltransferase activity
GO:0008609	alkyl-DHAP synthase activity
GO:0047105	4-trimethylammoniobutyraldehyde dehydrogenase activity
GO:0004961	TXA(2) receptor activity
GO:0047787	delta4-3-oxosteroid 5beta-reductase activity
GO:0003991	acetylglutamate kinase activity

Although bidirectional promoters are orthologous between humans and mice, they exhibit sparse conservation signals in multi-species alignments. This is a slightly surprising result, given that sequence conservation is a reliable marker for functional elements. Nevertheless, it is possible that alternative methods may reveal similarities in bidirectional promoters across species.

To test for similarity in sequence characteristics that may reveal subtle similarities between the sets of human and mouse bidirectional promoters, we calculated a log-likelihood score called Regulatory Potential (RP). The RP score was used in ESPERR (Evolutionary and Sequence Pattern Extraction through Reduced Representations) [10] to capture information in sequence alignments over seven vertebrate species. This method has been shown to discriminate regulatory regions from nonfunctional regions with an accuracy of 80% [10].

**Figure 3:**
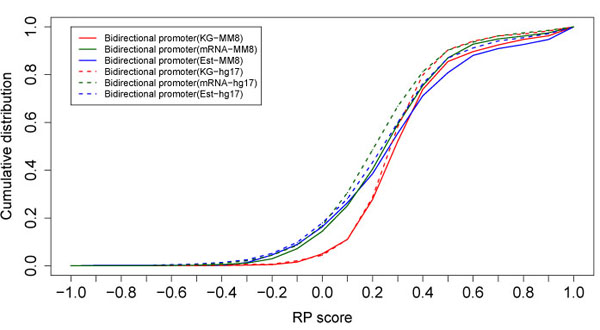
RP score cumulative distribution functions for bidirectional promoters in human and mouse. Bidirectional promoters identified from Known Genes (KG), mRNA, and ESTs all yield similar scores in both human and mouse genomes. RP scores were calculated based on genome assemblies hg17 (human) and mm8 (mouse).

The RP score cumulative distribution functions plotted in Figure 3 reveal that regulatory potential scores are similar for bidirectional promoters defined by Known Genes, ESTs, and mRNA in both human and mouse. The similarity in profiles exhibited by all three datasets for each species indicates that sequence characteristics are similar in bidirectional promoter regions, both across species (human vs. mouse) and across datasets (Known Genes, mRNA, and ESTs). The strategy used to map these gene pairs across species strongly identifies orthologous genes that are characterized by name. Therefore the conclusions should not change as more data is added.

### Discriminating functional elements based on RP scores

Having established the orthology of bidirectional promoters between human and mouse, we now shift our attention to the problem of discriminating functional elements in the human genome. We again make use of RP scores, which have proven useful for discriminating functional elements from nonfunctional elements, yet their ability to discriminate among types of functional elements remains unknown.

To test the hypothesis that sequence characteristics differ between classes of functional elements, thereby allowing these classes to be discriminated, we compared RP scores for human bidirectional promoters to those for other functional regions, including enhancers, unidirectional promoters, unbounded promoters, non-promoters (i.e. tail-to-tail regions), coding regions, and neutral regions.

**Figure 4:**
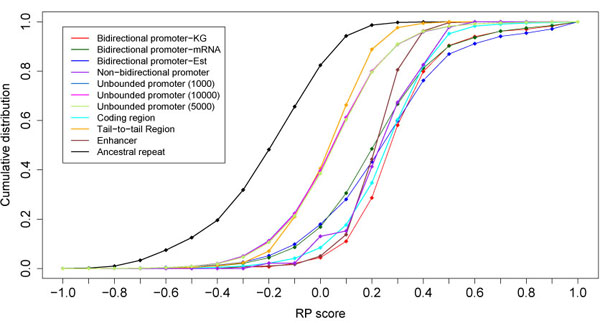
Cumulative distribution functions of RP scores for different functional classes. These include bidirectional promoters (red, green, blue), non-bidirectional promoters (purple) and unbounded promoters (light blue, pink, light green). Other functional elements are coding regions (aqua), tail-to-tail regions (yellow) and enhancers (maroon). The nonfunctional elements are represented by ancestral repeats (black).

The cumulative distribution functions of RP score for the different functional classes are shown in Figure 4. We observe that:

• As expected, neutral regions (represented by ancestral repeats) separated very distinctly from functional regions such as enhancers.

• Despite the fact that bidirectional promoters do not have a strong signal for sequence conservation, they have slightly higher RP scores than enhancers. This is significant because the enhancers used in this analysis are enhancers of genes involved in essential developmental processes, such as neurogenesis [11], which are characterized by strong signals of sequence conservation known as Multi-species Conserved Sequences (MCSs) [12].

• Bidirectional promoters have high RP scores, similar to unidirectional promoters, which are promoter regions that are defined by two genes in a head-to-tail configuration. Like bidirectional promoters, unidirectional promoters are bounded on both sides by exons.

• High scores are not a feature of all promoter regions. For example, unbounded promoters, which are promoters having no neighboring upstream gene, tend not to have high RP scores. We examined unbounded promoter regions with no upstream gene within 1000, 5,000, and 10,000 bp and found moderately low RP scores for all three classes. Furthermore, the range of these scores was indistinguishable from non-promoter regions.

• Coding regions score nearly as well as bidirectional promoters. This suggests that the types of nucleotide substitutions and the “word” content of bidirectional promoters and coding regions may be governed by the same rules, despite that fact that coding regions are strongly conserved and bidirectional promoters are not.

### Prediction of bidirectional promoters from RP scores

On the basis of Figure 4, it is apparent that bidirectional promoter regions tend to have higher RP scores than either non-promoter or unbounded promoter regions. Another way to see this is to plot the class-conditional density functions *p*(*x*|*C*), where *x* is the RP score, and *C* is a functional class; this is simply the probability density function of RP scores, restricted to the functional class *C*. Given the class-conditional density functions *p*(*x*|*C*_1_) and *p*(*x*|*C*_2_) for classes *C*_1_ and *C*_2_, respectively, we can construct a likelihood ratio classifier that maps an RP score *x* to a functional class using the rule:

If p(x|C1)p(x|C2) {> μ Decide class C1< μ Decide class C2

The performance of this classifier for different values of the threshold μ is summarized by a Receiver Operating Characteristic (ROC), which is a plot of sensitivity against (1—specificity). We constructed two such classifiers: one to discriminate bidirectional promoters from non-promoters, and the other to discriminate bidirectional promoters from unbounded promoters.

#### Distinguishing bidirectional promoters from non-promoters

We constructed a likelihood-based classifier to distinguish bidirectional promoters from non-promoters; this is a two-class classification problem, in which the two classes are:

$$\begin{array}{l} C_{1}=\;\{\text{bidirectional promoters}\}\\ C_{2}\;=\;\{\text{non-promoters\}} \end{array}$$

**Figure 5:**
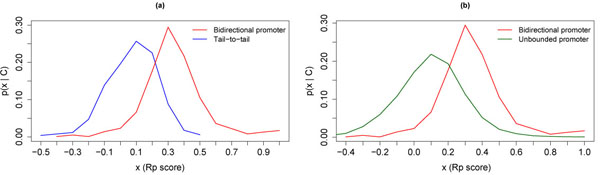
(a) Class-conditional probability density functions *p*(*x*|BP) (bidirectional promoters) and *p*(*x*|NP) (non-promoters). (b) Class-conditional probability density functions *p*(*x*|BP) (bidirectional promoters) and *p*(*x*|UBP1000) (unbounded promoters).

**Figure 6:**
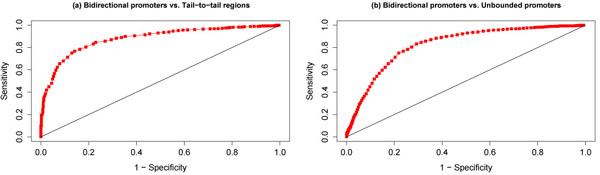
(a) Receiver operating characteristic (ROC) for classifier that discriminates bidirectional promoters from non-promoters. (b) Receiver operating characteristic (ROC) for classifier that discriminates bidirectional promoters from unbounded promoters.

The class-conditional probability distributions *p*(*x*|BP) and *p*(*x*|NP) are shown in Figure 5(a) (here “BP” denotes the class of bidirectional promoters, and “NP” denotes the class of non-promoters). The corresponding ROC curve is shown in Figure 6(a). A Maximum Likelihood classification rule (obtained by setting μ = 1 in the likelihood ratio classifier (1)) yielded a test set accuracy of 74%, a specificity of 92% (relatively high), and a sensitivity of 65% (relatively low), as shown in Table 3. The ROC curve reveals that the sensitivity can be boosted above 80% by trading off for a specificity below 80%.

**Table 3:** Performance of classifiers on test data

Classifier	Accuracy (%)	Sensitivity (%)	Specificity (%)
Bidirectional promoter vs. Non-promoter	74.54	65.53	92.16
Bidirectional promoter vs. Unbounded promoter	80.37	67.94	81.10

#### Distinguishing bidirectional from unbounded promoters

We constructed a likelihood-based classifier to distinguish bidirectional promoters from unbounded promoters (specifically, the class of promoters with no upstream gene within 1000 base pairs); this is a two-class classification problem, in which the two classes are:

$$\begin{array}{l} C_{1}\;=\;\{\text{bidirectional promoters\}}\\ C_{2}\;=\;\{\text{unbounded promoters}\;\text{(1000}\;\text{bp)\}} \end{array}$$

The class-conditional probability distributions *p*(*x*|BP) and *p*(*x*|UBP1000) are shown in Figure 5(b) (here “BP” denotes the class of bidirectional promoters, and “UBP1000” denotes the class of promoters with no upstream gene within 1000 base pairs). The corresponding ROC curve is shown in Figure 6(b). A Maximum Likelihood classification rule (obtained by setting μ = 1 in the likelihood ratio classifier (1)) yielded a test set accuracy of 80%, a specificity of 81% (relatively high), and a sensitivity of 67% (relatively low), as shown in Table 3. The ROC curve reveals that the sensitivity can be boosted above 80% by trading off for a specificity below 75%.

### Multiple Class Prediction

We then tackled a more challenging problem—to construct a classifier that distinguishes the following four classes:

$$\begin{array}{l} C_{1}=\{\text{bidirectional promoters}\}\\ C_{2}=\{\text{unbounded promoters (1000 bp)}\}\\ C_{3}=\{\text{enhancers}\}\\ {\mathrm{C4}}_{}=\{\text{non-promoters}\} \end{array}$$

**Figure 7:**
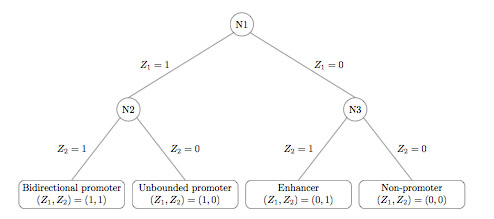
Algorithm for classifying regions into one of four classes: bidirectional promoter, unbounded promoter, non-promoter, or enhancer.

It turns out that bidirectional promoters and unbounded promoters are enriched in CpG islands, while enhancers and non-promoters are depleted in CpG islands. Furthermore, bidirectional promoters and enhancers tend to have relatively high RP scores as compared to unbounded promoters and non-promoters. It follows that by making use of both features (presence of CpG islands and RP score), we may be able to separate the four classes. We therefore implemented a two-stage hierarchical classifier (Figure 7). The first stage only looks at the CpG island feature: if CpG islands are present, the instance is passed to the left child at level 2 (node N2), while if CpG islands are not present, the instance is passed to the right child at level 2 (node N3). There is also a classification outcome *Z*_1_ of the first stage; if the instance was passed to the left child, then *Z*_1_ = 1, else *Z*_1_ = 0. Ideally, instances that end up in node N2 should be either bidirectional or unbounded promoters, while instances that end up in node N3 should be either enhancers or non-promoters. The next stage of the classifier then refines the classification further. Node N2 uses a support vector machine to separate bidirectional from unbounded promoters based on two features—the presence of CpG islands and RP score, while node N3 uses a decision tree to separate enhancers from non-promoters based on one feature—RP score (it turns out that these two classes cannot be distinguished based on the presence of CpG islands, so this feature would not be helpful). A decision tree was used at node N3 because it gave better results that a support vector machine. There is a classification outcome *Z*_2_ associated to each node at level 2. For node N2, *Z*_2_ = 1 implies that the instance is classified as a bidirectional promoter, while *Z*_2_ = 0 implies that the instance is classified as an unbounded promoter. For node N3, *Z*_2_ = 1 implies that the instance is classified as an enhancer, while *Z*_2_ = 0 implies that the instance is classified as a non-promoter. The overall classification is then given by the pair (*Z*_1_, *Z*_2_) as follows:

**Table:** 

Class	(*Z*_1_, *Z*_2_)
Bidirectional promoters	(1,1)
Unbounded promoters	(1,0)
Enhancers	(0,1)
Non-promoters	(0,0)

## Conclusions

Bidirectional promoters aid in the analysis of promoter regions, as they are bounded on both sides by other functional elements, and thus precisely delineate the promoter region. Moreover, despite a lack of strong sequence conservation, bidirectional promoters exhibit conserved structure across species, which will undoubtedly be helpful in tracing evolutionary and species-specific events.

Predictive approaches to classifying functional elements in the human genome are frequently based on a variety of experimental characteristics (e.g. [13,14]). Here we have demonstrated that machine learning approaches can be effective without experimental data; this is the first evidence that different types of promoters can be discriminated from one another through machine learning approaches.

## Methods

Bidirectional promoters from the mouse genome were mapped to annotated transcripts in mouse assemblies mm5 and mm8 using the approach outlined in [3]. Comparison to CAGE data was accomplished by extracting all promoters from the RIKEN database and comparing genomic coordinates (from the assembly mm5). Any coordinates within 50 bp of each other on the same strand of DNA were considered to be a match. RP scores were collected over the range of each functional element using tools developed by David King of Penn State University (manuscript in preparation). Scores are available for the mouse mm8 assembly. Conserved occurrences of bidirectional promoters were identified by mapping the gene name from human to mouse and searching the Known Gene annotations for the 5′ end of a neighboring gene that falls within 1000 bp.

From the Known Gene track of the human genome, we identified approximately 1006 bidirectional promoters, 525 non-promoters, 275 enhancers, and over 15,000 unbounded promoters. This data was used to train and test both our two-class classifiers and our four-class classifier.

The accuracy, sensitivity, and specificity values for the two-class case (Table 3) were calculated using:

$$\begin{array}{c} \text{Overall Accuracy =}\;\frac{N_{11}\;+N_{22}}{\sum_{i=1}^{2}\;\sum_{j=1}^{2}\;N_{ij}\;}\\ \text{Sensitivity =}\;\frac{N_{11}}{\sum_{i=1}^{2}\;N_{i1}}\\ \text{Specificity}\;\text{=}\;\frac{N_{22}}{\sum_{i=1}^{2}\;N_{i2}} \end{array}$$

where *N_ij_* be the number of class *C_j_* instances classified to class *C_i_* and for the purpose of calculating sensitivity and specificity we have taken the positive class to be *C*_1_ and the negative class to be *C*_2_.

For the four class case (Table 4), the overall accuracy and the accuracy over a specific class are given by

$$\begin{array}{l} \text{Overall Accuracy =}\;\frac{\sum_{i=1}^{4}\;N_{ii}}{\sum_{i=1}^{4}\;\sum_{j=1}^{4}\;N_{ij}\;}\\ \text{Accuracy over class }C_{j}\text{ =}\;\frac{N_{jj}}{\sum_{i=1}^{4}\;N_{ij}\;} \end{array}$$

**Table 4:** Performance of four-class hierarchical classifier based on three-fold cross-validation

Class	(*Z*_1_, *Z*_2_)	Accuracy (%)
Bidirectional promoters	(1,1)	71.31
Unbounded promoters	(1,0)	62.26
Enhancers	(0,1)	66.13
Non-promoters	(0,0)	81.41
Overall		70.56

By the way the four-class hierarchical classifier is constructed, any promoters lacking CpG islands will be diverted down the left child of node N1, and thus will be misclassified. It follows that the performance of the algorithm is acutely sensitive to the fraction of promoters with CpG islands in the test set. Since it is known that CpG islands are present in roughly 70% of promoters, we constructed our test set using a stratified sampling approach that guaranteed that 70% of promoters in the test set contained CpG islands; this helped to reduce the variation in the performance due to sampling.

## Competing interests

The authors declare that they have no competing interests.

## Authors' contributions

LE conceived of the study. MQY implemented the software and performed the analyses. Both authors contributed to writing the manuscript.
